# Epidemiology of Pathogens Listed as Potential Bioterrorism Agents, the Netherlands, 2009‒2019

**DOI:** 10.3201/eid2907.221769

**Published:** 2023-07

**Authors:** Jorrit Broertjes, Eelco Franz, Ingrid H.M. Friesema, Hugo-Jan Jansen, Frans A.G. Reubsaet, Saskia A. Rutjes, Cornelis Stijnis, Bettie C.G. Voordouw, Maaike C. de Vries, Daan W. Notermans, Martin P. Grobusch

**Affiliations:** Amsterdam University Medical Centers, Amsterdam the Netherlands (J. Broertjes, C. Stijnis, M.P. Grobusch);; National Institute of Public Health and the Environment, Bilthoven, the Netherlands (E. Franz, I.H.M. Friesema, F.A.G. Reubsaet, S.A. Rutjes, C.B.C.G. Voordouw, M.C. de Vries, D.W. Notermans);; Ministry of Defence, Doorn, the Netherlands (H.-J. Jansen);; Royal Netherlands Navy, Den Helder, the Netherlands (C. Stijnis)

**Keywords:** epidemiology, pathogens, bacteria, viruses, diseases, biosecurity, potential bioterrorism agents, bioterrorism and preparedness, the Netherlands

## Abstract

We provide incidences (cases/10 million persons) in the Netherlands during 2009–2019 for pathogens listed as potential bioterrorism agents. We included pathogens from the highest categories of the European Medicines Agency or the US Centers for Disease Control and Prevention. Notifiable diseases and recently published data were used to calculate the average annual incidence. *Coxiella burnetii* had the highest incidence because of a Q fever epidemic during 2007–2010. Incidence then decreased to 10.8 cases/. Pathogens with an incidence >1 were *Brucella* spp. (2.5 cases), *Francisella tularensis* (1.3 cases), and *Burkholderia pseudomallei* (1.1 cases). Pathogens with an incidence <1 were hemorrhagic fever viruses (0.3 cases), *Clostridium botulinum* (0.2 cases), and *Bacillus anthracis* (0.1 cases). Variola major and *Yersinia pestis* were absent. The generally low incidences make it unlikely that ill-meaning persons can isolate these pathogens from natural sources in the Netherlands. However, the pathogens are stored in laboratories, underscoring the need for biosecurity measures.

An act of bioterrorism could affect public health and will cause substantial societal disruption. Although the risk for bioterrorism is considered to be low, an incident involving a limited number of persons, or even a hoax, may result in panic in the general public, and a larger attack might have major consequences up to total disruption of society (*1*). Despite the low probability, the risk for bioterrorism remains and should not be neglected. The continued interest of terrorists in bioweapons and toxins is illustrated by the 2018 foiled plot of a ricin attack by an extremist from Tunisia in Cologne, Germany (*2*) and a ricin letter sent in 2020 to the President of the United States (*3*). The continued risk for bioweapons was addressed during the Munich Security Conference in 2018 by the Dutch Minister of Defense (*4*). Furthermore, the US government designates ﻿biologic weapons as a persistent threat in their 2022 National Biodefense Strategy (*5*).

The pathogens listed as potential bioterrorism agents originate from nature and, although rare, can be encountered either as causing autochthonous or travel-related disease. The aim of this study was to provide an overview of the incidences of these pathogens, exemplified by the Netherlands, to maintain awareness for biosafety at laboratories, and to underscore the relevance of material accountability and the need for biosecurity measures to prevent unauthorized access to the actual pathogens and related knowledge, to reduce the risk for bioterrorism.

There are many pathogens that could potentially be used as bioweapons based on characteristics such as route of transmission, pathogenicity, infectious dose, stability in the environment, and other factors (*6*). It is essential to reduce the risk for misuse and deliberate release by controlling and restricting the development, production, stockpiling, or other ways of acquiring biologic and toxin weapons or their means of delivery. Several lists have been compiled to guide measures to mitigate the risks regarding these pathogens (Table 1).

**Table 1:** Overview of lists related to biosafety and the deliberate release of pathogens*

Organization	List	Purpose	Reference
Australia Group	Handbook volume II	Nonproliferation	(*7*)
Biosecurity office, RIVM	Combined list of biologic agents	Comparison of lists relevant for the Netherlands	(*8*)
CDC	Biosafety in Microbiological and Biomedical Laboratories	Biosafety	*(**9*)
	Bioterrorism Agents/Diseases	Bioterrorism preparedness	(*10**,**11*)
	Classification of Diseases, Functioning, and Disability	Natural environment protection (GMO regulations)	(*12*)
	Federal Select Agent Program	State biosecurity legislation	(*13*)
EMA	Biologic and Chemical Threats	Bioterrorism preparedness	(*14*)
	Guidance document on use of medicinal products for the treatment and prophylaxis of biologic agents that might be used as weapons of bioterrorism	Civilian or military medical guidelines for biowarfare/bioterrorism	(*15*)
European Union	Biologic agents directive 2000/54/EC	Biosafety	(*16*)
	Regulation 2017/2268 dual-use items	Nonproliferation	(*17*)
German Federal Ministry of the Interior	War Weapons Control Act	Nonproliferation	(*18**,**19*)
NATO	Handbook on the Medical Aspects of NBC Defensive Operations	Civilian or military medical guidelines for biowarfare/bioterrorism	(*20*)
USAMRIID	Medical Management of Biologic Casualties Handbook	Civilian or military medical guidelines for biowarfare/bioterrorism	(*21*)

*EMA classification of biologic threats adapted from EMA. The encephalitis viruses are Eastern equine encephalitis, Western equine encephalitis, and Venezuelan equine encephalitis. Other hemorrhagic fever viruses, for which no treatment existed: Marburg virus, Ebola virus, yellow fever virus, hantavirus, and tick-borne encephalitis virus. However, for Ebola virus disease, treatment options now exist. CDC, US Centers for Disease Control and Prevention; EMA, European Medical Agency; NATO, North Atlantic Treaty Organization; NBC, nuclear, biological, chemical; RIVM, National Institute for Public Health and the Environment, the Netherlands; USAMRIID, US Army Medical Research Institute of Infectious Diseases.

Only 2 of the lists are directly devoted to bioterrorism: the US Centers for Disease Control and Prevention (CDC) Bioterrorism Agents/Diseases and the European Medicines Agency (EMA). A prioritization and selection of the potential bioterrorism agents was made by CDC 2 decades ago, which resulted in the Bioterrorism Agents/Diseases classification (*10*). This classification is based on public health experience, as well as Cold War era military experiments, in which potential bioterrorism agents were evaluated for public health impact, dissemination possibilities, public perception, and the need for special preparation (*11*). The EMA restructured the CDC list, creating the Biologic and Chemical Threats list, of which the biologic agents are provided (Table 2) (*12*). The 3 categories are created from a medical point of view and accompanied by treatment guidelines (*15*).

**Table 2:** European Medicines Agency classification of biologic threats

Category I agents: major infectious diseases for which treatment exists	Category II agents: other bacterial infections for which treatment exists	Category III agents: biologic agents for which currently no specific treatment can be recommended
*Bacillus anthracis, Yersinia pestis, Francisella tularensis,* Variola major, hemorrhagic fever viruses, botulinum toxin (*Clostridium botulinum*), *Brucella* spp., *Coxiella burnetii*, *Burkholderia mallei*, *Burkholderia pseudomallei*	*Chlamydia psittaci, Rickettsia prowazekii, Mycobacterium tuberculosis, Shigella* spp., *Salmonella* spp., *Vibrio cholerae*	Enterohemorrhagic *Escherichia coli, Cryptosporidium,* encephalitis viruses,* Nipah virus, other hemorrhagic fever viruses,† *Clostridium perfringens* epsilon toxin, Staphylococcal enterotoxin B, ricin

*Encephalitis viruses include Eastern equine encephalitis, Western equine encephalitis, and Venezuelan equine encephalitis. †Other hemorrhagic fever viruses, for which no treatment existed: Marburg virus, Ebola virus, yellow fever virus, hantavirus, and tick-borne encephalitis virus. However, for Ebola virus disease, treatment options now exist.

## Materials and Methods

### Selection of Potential Bioterrorism Agents

This study includes the pathogens that are categorized in the highest risk category by either EMA (Table 2) or CDC. The CDC category A contains the classic potential bioterrorism agents: *Bacillus anthracis, Yersinia pestis, Francisella tularensis*, *Clostridium botulinum* toxin, and hemorrhagic fever viruses (*10*). EMA category I includes the same pathogens, expanded by *Brucella* spp., *Burkholderia mallei* and *Burkholderia pseudomallei*, and *Coxiella burnetii* (*14*).

### Nationally Notifiable Diseases and EMA Major Biologic Threats

Many potential bioterrorism agents are notifiable diseases in the Netherlands because of their infectiousness and virulence. Notifications of these diseases are collected at the National Institute for Public Health and the Environment (RIVM), and data are made publicly available (*22*). Not all potential bioterrorism agents from the highest categories are notifiable diseases in the Netherlands. Biologic threats that are not notifiable are *B. mallei* and *B. pseudomallei*, the causative agents of glanders and melioidosis, respectively. An overview of melioidosis cases in the Netherlands was recently published by Birnie et al. (*23*). Furthermore, Rijks et al. recently published the incidence of *F. tularensis* in the Netherlands (*24*). These data were added to the overview of the major biologic threats.

### Observation Period

The observation period was set from 2009 through 2019. January 2009 was selected as the starting point because in 2008 the Netherlands implemented the Public Health Act, on which the current selection of nationally notifiable diseases and notification criteria are based (*25*). December 2019 was selected as the endpoint because measures taken to combat the SARS-CoV-2 pandemic in 2020 and 2021, including limiting international travel, mitigated the incidence of many other infectious diseases. Because the aim of this study was to provide an overview of the standard incidence, we excluded those years.

### Calculating Average Annual Incidences Per 10 Million Persons

To enable international comparison, we calculated the average annual incidences per 10 million persons. During the observation period, the population of the Netherlands increased from 16.5 million in 2009 to 17.3 million in 2019 (*26*). We calculated average incidence per year and, subsequently, the average annual incidence.

## Results

We compiled absolute numbers of cases and annual incidence for each organism (Table 3; Figure). Detailed descriptions of cases and incidence for individual pathogens follow.

**Table 3:** Epidemiology of pathogens listed as highest category potential bioterrorism agents, the Netherlands, 2009–2019*

Pathogen	2009	2010	2011	2012	2013	2014	2015	2016	2017	2018	2019	Average annual incidence
*Bacillus anthracis*	0	0	0	0	0	0	0	0	0	2	0	0.1
*Brucella* spp.	4	6	1	2	5	2	9	4	2	5	7	2.5
*Burkholderia mallei*	0	0	0	0	0	0	0	0	0	0	0	0.0
*Burkholderia pseudomallei*	0	2	0	5	2	1	1	1	5	2	NA	1.1
*Clostridium botulinum*	0	0	0	2	0	0	0	2	0	0	0	0.2
*Coxiella burnetii*	2,424	411	77	63	20	26	20	14	22	18	18	171.2 (10.8)†
*Francisella tularensis*	1	0	1	0	1	3	1	9	2	2	4	1.3
Variola major	0	0	0	0	0	0	0	0	0	0	0	0.0
*Yersinia pestis*	0	0	0	0	0	0	0	0	0	0	0	0.0
Hemorrhagic fever viruses	0	0	0	0	0	1‡	0	0	1§	2§	2¶	0.3

*Incidence is given as cases/10 million persons. NA, not available. †Incidence is shown in parentheses for postoutbreak years of 2015–2019, which is more representative. ‡Ebola fever virus. §Yellow fever virus. ¶Lassa fever virus.

**Figure:**
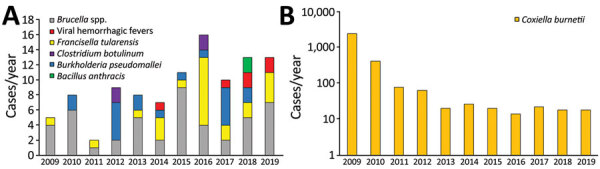
Infections with pathogens listed as potential bioterrorism agents in the Netherlands, 2009−2019. A) Absolute numbers for most pathogens. No cases of infection with *Burkholderia mallei*, variola major virus (smallpox), or *Yersinia pestis* were reported. B) *Coxiella burnetii* is shown on a logarithmic scale to accommodate the high incidence during the Q fever epidemic of 2007‒2010. Complete data are shown in Table 2.

### B. anthracis

The incidence of *B. anthracis* ranged from 0 to 2 cases/year during 2009–2019 in the Netherlands. This finding results in an average annual incidence for *B. anthracis* of 0.1 cases/10 million persons. Two cases of the cutaneous form of anthrax occurred in 2018. One patient was a returning traveler from Tanzania who had no possible source of infection. The second patient had a travel history to Turkey and possibly got infected during the slaughtering of sheep or preparation of meat. No anthrax spores have thus far been detected in hoax letters in the Netherlands (*27*).

### *Brucella* spp.

Brucellosis occurred only as an imported disease in the Netherlands (1‒9 cases/year). The average annual incidence during 2009–2019 of *Brucella* spp. was 2.5 cases/10 million persons.

### B. mallei

Glanders is not a notifiable disease in the Netherlands; therefore, no data are available on *B. mallei* cases. However, no *B. mallei* has been detected in patient samples or cultured isolates received by RIVM for confirmation of rare or highly virulent pathogens, and no cases were otherwise reported during 2009–2019 (RIVM, unpub. data). Furthermore, no scientific publications were identified describing cases of glanders in the Netherlands.

### B. pseudomallei

Melioidosis is also not a notifiable disease in the Netherlands. However, Birnie et al. identified 33 returning travelers who had this disease over the past 25 years (*23*). During 2009–2019, the average annual incidence was 1.1 cases/10 million persons (range 0‒5 total cases/year) in the Netherlands.

### *C. botulinum* Toxin

The incidence of botulism ranged from 0 to 2 cases/year, resulting in an average annual incidence of 0.2 cases/10 million persons. In 2012, two unrelated cases of infant botulism were reported, 1 possibly related to honey consumption and 1 that did not have a suspected source (*28*). In 2016, two additional case-patients were given diagnoses of autochthonously acquired botulism in the Netherlands. The first case-patient was a young man from eastern Europe who had no confirmed exposure. The second case-patient had suspected foodborne botulism, possibly related to the use of glass jars for home preservation of food, which is associated with botulism (*29*).

### C. burnetii

During 2007‒2010, an epidemic of Q fever occurred in the Netherlands that was related to goat farming and had a peak of 2,424 cases in 2009. This epidemic was the largest recorded outbreak globally (*30*). Since then, preventative measures have been taken, and the annual incidence has decreased to preoutbreak levels. For the total 2009–2019 period, the average annual incidence of *C. burnetii* was 171.2 cases/10 million persons. However, during the last 5 years of the study period (2015–2019), the average incidence was 10.8 cases/10 million persons (14 to 26 cases/year).

### F. tularensis

For decades, *F. tularensis* cases did not occur in the Netherlands. However, sporadic imported cases were seen, such as a single imported case from Finland in 2009 (*31*). However, in 2011, an autochthonous case of *F. tularensis* was detected for the first time since 1953. The disease became a notifiable disease in 2016, and an overview of all 26 autochthonous cases from 2011–2021 has been published (*24*). A total of 21 autochthonous cases were reported during 2011–2019. There were 2 imported cases in 2019 (RIVM, unpub. data), resulting in 24 cases during 2009–2019 (range 0‒9 total cases/year). The annual average incidence of *F. tularensis* was 1.3 cases/10 million persons. Those cases were all *F. tularensis* subspecies *holarctica* (type B), which is the type from Europe and rarely associated with severe disease. The more severe pathogenic *F. tularensis* subspecies *tularensis* (type A) from North America has not been found in the Netherlands (*24*).

### Hemorrhagic Fever Viruses

In 2014, a military peacekeeper from Nigeria who was given a diagnosis of Ebola was transferred to the Netherlands as part of an international agreement during the West Africa Ebola epidemic. The patient was given treatment at the Major Incident Hospital of the University Medical Centre of Utrecht and fully recovered from Ebola (*32**,**33*).

In 2017, yellow fever was diagnosed in a woman returning from Suriname at the University Medical Centre Groningen; she survived (*34*). In 2018, two cases of yellow fever were imported to the Netherlands. One case was in a woman returning from Brazil, who recovered (*35*). The second case was in a man returning from the Gambia‒Senegal region. He was admitted to the Amsterdam Medical Centre and referred to the Erasmus Medical Centre because of liver failure and for transplantation (*36*).

In 2019, Lassa fever was diagnosed in 2 repatriated physicians from Sierra Leone; 1 died (*37*). This finding resulted in an average annual incidence for hemorrhagic fever viruses of 0.3 cases/10 million persons.

### Variola Major Virus and *Y. pestis*

No cases of infection with variola major virus (smallpox) or *Y. pestis* occurred during 2009–2019 in the Netherlands. 

## Discussion

Pathogens from EMA category II and category III are excluded from this study because those pathogens are considered to pose lower risks from an intentional release perspective. The EMA modified the CDC list from both a bioterrorism risk, as well as from a medical point of view, accompanied by a treatment guideline. EMA category I does not include Marburg and Ebola viruses because no treatment was available for infection with these viruses when the classification was made. Currently, at least for Zaire Ebola virus at least, treatment options are available (*38*). Because these filoviruses are included in CDC Category A, they are included in this study.

This example indicates that the EMA classification might require updating. Furthermore, it could be argued that the European Centre for Disease Prevention and Control is a more logical institution to maintain the list of potential bioterrorism agents for the European Union.

The most common pathogen found in the Netherlands was *C. burnetii*, a zoonotic, gram-negative bacterium whose reservoirs are mainly goats, sheep, and other herbivores. Q fever is associated with intensive goat farming and is endemic to the Netherlands, which is the second-largest exporter of agricultural products in the world (*30*). During parturition, many spore-like bacteria are released, which remain viable in the environment for months. This pathogen is highly infectious; inhalation of as few as 10 aerosolized organisms can cause disease. Disease ranges from asymptomatic or mild to severe forms, such as pneumonia or endocarditis. Some patients show development of Q fever fatigue syndrome (*5*). The incidence was much higher than average during the 2007–2010 epidemic. Afterward, the incidence decreased to ≈10 cases/10 million persons/year. Doxycycline is the first-choice treatment because of obligate intracellular growth of *C. burnetii* (*15*,*39*).

Since 2011, *F. tularensis* subspecies *holarctica* has been endemic to the Netherlands. This pathogen has a terrestrial lifecycle associated with animals around water and mud. This pathogen is considered a potential bioweapon because it can be aerosolized and is highly infectious; a single bacterium can cause disease (*40*). It can enter through the ﻿skin, conjunctiva, oral, or lungs. The disease ranges from ulcers to potentially fatal pneumonia (*24*). Determination of the subspecies is essential; identifying *F. tularensis* subspecies *tularensis* in a patient in the Netherlands who had no history of travel to North America would be a trigger to further investigate the likelihood of a deliberate release. Because of the facultative intracellular nature of this pathogen, suitable antimicrobial drugs for treatment are gentamicin, doxycycline, and ciprofloxacin (*41*).

*Brucella* spp., in contrast to *F. tularensis*, are not endemic to the Netherlands and are encountered only as imported cases. *B. melitensis* and *B. abortus* can be found in different animal species (sheep, goats, cattle); *B. suis* is found in pigs and *B. canis* in dogs. Recently, a case of *B. canis* was reported in a dog breeder in the Netherlands (*42*). *Brucella* spp. are considered potential bioweapons and constitute a well-established cause of laboratory infections (*43*). The bacteria can be aerosolized and have a low infectious dose, ranging from 10 to 100 microorganisms (*44*). Disease ranges from mild disease to osteomyelitis and endocarditis. Brucellosis is treated with doxycycline plus gentamicin or rifampin or streptomycin (*29**,**45*).

Another travel-related pathogen is *B. pseudomallei*, found in soil of (sub-)tropical regions and considered a potential bioweapon because of the high mortality rate and possibility of aerosol formation (*46*). Empirical antimicrobial drug treatment regimens for pneumonia based on cefuroxime or ceftriaxone are not effective against this pathogen (*47*). Suitable antimicrobial drugs are ceftazidime or meropenem (*48*).

Hemorrhagic fever virus cases are sporadically imported to the Netherlands but can cause considerable safety concerns for health care providers. In Europe in general, imported cases of viral hemorrhagic fevers are relatively rare. For example, within the EuroTravNet sentinel surveillance network reporting on >100,000 cases of imported infectious diseases within its realm in the 20-year period from 1998 to 2018, just 44 cases of viral hemorrhagic fevers were recorded (*49*). For hemorrhagic fever viruses, antiviral treatment options are limited, other than supportive therapeutic measures. For Ebola, treatment options are available (*38*).

*C. botulinum* is a spore-forming, anaerobic, gram-positive, rod-shaped bacterium that can be ubiquitously found in the soil and agricultural products. Botulinum toxin in one of the most potent neurotoxins; it results in paralysis (*50*). Detection of botulinum toxin, alongside *C. botulinum* DNA, can be indicative of foodborne botulism, either unintentionally or as an unsophisticated attempt at bioterrorism. If toxins are found without *C. botulinum* or its DNA, especially if deployed by aerosolization, this finding would indicate deliberate release and technical sophistication (*51*). Treatment consist of botulism antitoxin only for wound botulism. Additional antimicrobial drug treatment is indicated, and suitable options are (benzyl-)penicillin or metronidazole (*52**,**53*).

The reservoir of *B. anthracis* is soil, and infection can occur through infected animals, ingestion of uncooked meat, or aerosols. *B. anthracis* has become increasingly rare because introduction of laws mandating the destruction of animal carcasses in 1942; only 9 cases have been reported in the Netherlands since 1976 (*27*). The low incidence in 2009–2019 is consistent with the incidence predating this period. However, anthrax spores in soil remain a potential risk. In 2013, *B. anthracis* DNA was detected in a so-called white pit near the city of Nijmegen; those old cattle graves, covered in quicklime, remain a potential source of anthrax (*54*). In addition, most natural cases are cutaneous or gastrointestinal anthrax, and only a small portion are pulmonary forms of anthrax. Bioterrorism would probably involve release of aerosolized spores; therefore, pulmonary anthrax is more indicative of intentional release. First-choice antimicrobial drugs are ciprofloxacin or doxycycline. Because of the high mortality rate for inhalation anthrax, treatment with multiple antimicrobial drugs is advised. Spores can have a long incubation time. Therefore, prolonged treatment is required. Furthermore, antitoxins and vaccines are available (*15**,**55*).

*B. mallei* is a potential bioterrorism agent that is not endemic to the Netherlands. Glanders is a disease primarily found in horses and was eradicated from the European Union by strict control measures. Infection can occur by contact with infected animals through mucous membrane or skin; clinical manifestations range from localized disease to sepsis (*56*). Similar to the case for *B. pseudomallei*, empirical antimicrobial drug treatment may not be effective for this pathogen; suitable antimicrobial drugs are ceftazidime or meropenem (*48*).

As expected, no cases of variola major infection were reported in 2009–2019 from the Netherlands. Variola major virus is highly infectious, and the infectious dose is 1 virus particle (*57*). It is also highly virulent, starting with an exanthema, which can progress to hemorrhagic lesions, and has a mortality rate <30%. The World Health Organization declared global eradication in 1980 because of a large-scale vaccination operation (*58*). The last smallpox epidemic in the Netherlands occurred in 1951 in the city of Tilburg, during which 51 infections occurred, resulting in 2 deaths (*59*). However, variola major stocks remain in 2 reference laboratories, designated by the World Health Organization: 1 in the United States at the CDC and 1 in Russia at the State Research Center of Virology and Biotechnology VECTOR. Although those laboratories have strict security measures, a release from the laboratory, either accidental or deliberate, is not unthinkable. Novel antiviral drugs are being developed (*60*,*61*). Furthermore, there are concerns about the possibility of resurrecting the virus by using synthetic biology (*62*). Finally, fragments of smallpox virus are believed to be present in the thawing Arctic permafrost. This possibility could pose additional, unknown risks (*63*).

Recently, a major outbreak of mpox (formerly known as monkeypox) was reported in most Western countries (*64*,*65*). Exceptions excluded, those cases typically were not characterized by a travel history to disease-endemic regions and resulted from local transmission, mainly in the men who have sex with men community (*66*). Mpox is not listed a potential bioterrorism agent by EMA or CDC. It is yet unclear whether this virus, originally a zoonosis from the family Poxviridae and related to variola virus, might be used as a biologic threat. A vaccine is available and proven to be effective to prevent mpox cases (*67*).

No cases of *Y. pestis* were found in the observation period. This finding was expected because the last human case of plague in the Netherlands was recorded in 1929 (*68*). However, *Y. pestis* continues to be endemic to animal reservoirs around the world and still causes epidemics, such as during 2017 in Madagascar (*69*). This infection can occur in a bubonic or pneumonic form. Infection can because of the bite of an infected flea by aerosols for pneumonic plague. The mortality rate for plague is 5%–15% if adequately treated and 50%‒90% if untreated (*70*). A recent update found ciprofloxacin to be a first-choice treatment (*71*).

Most pathogens in the top risk categories only occur sporadically in the Netherlands, restricting access by ill-meaning persons. However, some of the pathogens are present in clinical microbiology laboratories. Therefore, those pathogens must have proper systems in place that cover the key areas of biosecurity to minimize the risk for misuse, such as a policy on personnel and information security, material accountability, and physical security, as well as biosecurity awareness. Several assessment tools exist to assess the level of biosecurity implementation within an organization, such as the RIVM self-scan toolkit (*72*) and the more extended vulnerability scan (*73*), which provide questions, scenarios, and best practices built around the key areas of biosecurity.

Restrictive access to those pathogens is much less the case for biologic toxins such as ricin and abrin, which can be extracted from the seeds of the castor bean and the rosary pea plant, respectively; both are obtainable more easily than the bacteria or viruses described. The continued interest in biologic toxins is illustrated by a ricin attack prevented in 2018 in Cologne, Germany (*2*). Preventing this type of event requires the attention of law enforcement, customs agencies, and healthcare professionals.

Preparedness for an act of bioterrorism requires cross-sectoral collaboration, involving public health, law enforcement, and intelligence. The ability to detect and confirm individual cases of disease, in and of itself, is not proof of preparedness for a biologic attack or unusual unintentional outbreak. A high index of suspicion, clinical astuteness, and rapid epidemiologic and laboratory investigations by a robust, standing public infrastructure are required. The determination whether a pathogen was intentionally released ultimately rests with law enforcement agencies.

Despite the assumed low probability of an attack with biologic agents, it is essential to maintain (inter-)national preparedness. As noted, in 2018 in Cologne, Germany, an attempt to use ricin in an attack was prevented (*2*). This incident demonstrated the persistent interest for the use of biologic agents by perpetrators. Therefore, hospitals and medical microbiology laboratories should have up-to-date protocols on how to respond to biothreat agents. Methods that describe how to screen for biologic agents in suspected objects, such as powder letters, are included in the Dutch Chemical, Biologic, Radiological and Nuclear protocols (*74*).

The SARS-CoV-2 pandemic shows the potential of societal disruption caused by infectious diseases. For a resilient society, it is essential to maintain and improve the preparedness to not only natural events but also with the intentional release of biologic agents. This preparedness should be integrated in the well-established Chemical, Biologic, Radiological and Nuclear framework and through national and international cooperation.
